# Double fetus-in-fetu: CT scan diagnosis in an adult

**DOI:** 10.4103/0971-3026.54890

**Published:** 2009-08

**Authors:** BV Daga, VA Chaudhary, Amit S Ingle, Vinayak B Dhamangaokar, Deepti P Jadhav, Prasanna A Kulkarni

**Affiliations:** Department of Radiodiagnosis and Imaging Sciences, Dr. Vaishampaiyan Memorial Government Medical College, Solapur, Maharashtra, India

**Keywords:** Fetus-in-fetu, Computed Tomography

## Abstract

A fetus-in-fetu (FIF) is an uncommon developmental abnormality characterized by a reasonably well-formed but aborted fetus that is seen in the form of an encapsulated, pedunculated vertebrate tumor in the patient's abdomen. We report an interesting case of a double FIF in a 20-year-old man, who presented with acute abdominal pain and a lump. CT scan of the abdomen revealed two ill-formed fetuses-in-fetu, which were seen as a 15-cm complex, encapsulated mass in the lower retroperitoneum; there was also free fluid in the abdomen. The diagnosis of a ruptured twin FIF was made. Complete surgical excision of the lesion was performed and the diagnosis was confirmed histopathologically. To the best of our knowledge, the CT scan findings of a ruptured double FIF in an adult have not been previously documented.

## Introduction

Fetus-in-fetu (FIF) is a rare condition in which an encapsulated fetiform calcified mass is present in the abdomen of its host. It is supposed to be a highly differentiated form of teratoma.[1] However, in view of the fact that body parts can be identified within it, there is a tendency to consider this condition as being distinct from a teratoma. It has been suggested that if spinal elements are absent, the lesion is a teratoma, whereas if they are present the tumor can be considered to be a FIF.[1] We describe the case of a twin FIF that was diagnosed on CT scan.

## Case Report

A 20-year-old male presented in the emergency department with acute abdominal pain and distension. He had a history of a palpable abdominal lump since childhood. He was hemodynamically stable and the laboratory parameters were within normal limits. Abdominal examination revealed a tender, firm-to-hard palpable lump in the hypogastrium. Abdominal USG showed a complex mass in the lower abdomen, containing nodular soft tissue components and multiple echogenic areas with post-acoustic shadows. Based on these findings a provisional diagnosis of retroperitoneal teratoma was made. Alpha fetoprotein and beta-human gonadotropin hormone assays were not obtained.

**Figure 1:**
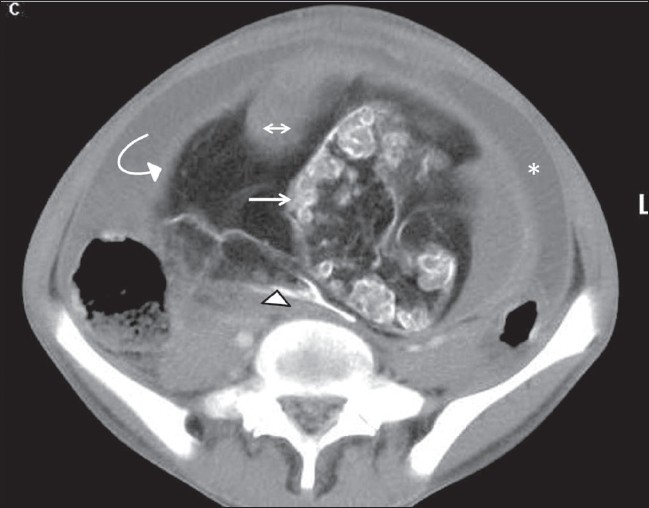
Axial contrast-enhanced CT scan shows the spine (arrow) and sacrococcyx (arrowhead) of the bigger fetus surrounded by fat and enclosed in a capsule (curved arrow). A soft-tissue plug is seen (double arrow). Ascites (*) is also seen

**Figure 2:**
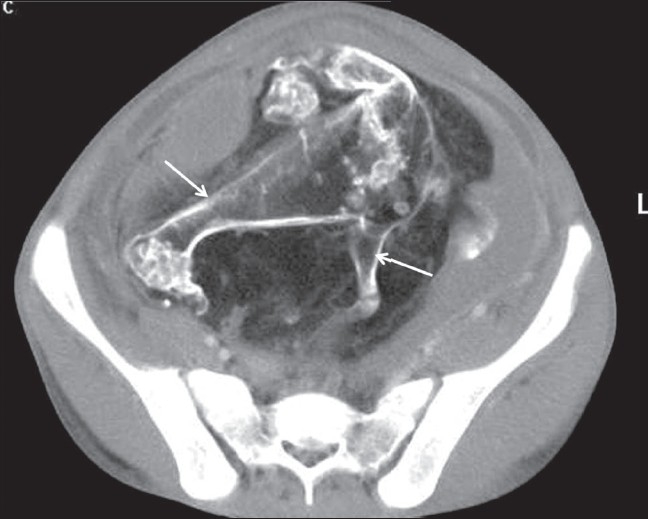
Axial contrast-enhanced CT scan shows ossified extremities (arrows) of the bigger fetus

**Figure 3:**
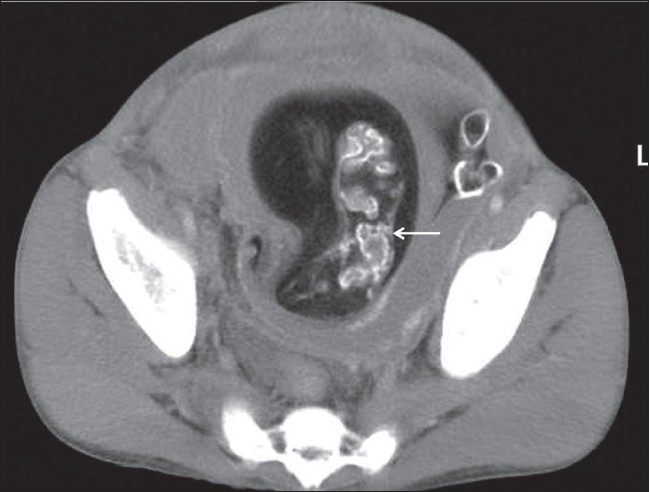
Axial contrast-enhanced CT scan (inferior to B) shows the rudimentary spine (arrow) of the smaller fetus

Abdominal CT scan was performed for further evaluation and this showed a lobulated complex mass in the retroperitoneum in the midline; the mass contained two separate sets of spinal elements, two sets of rudimentary pelves, sacra, extremity bones, phalanges, and other osseous elements surrounded by fat, all enclosed in a common sac. Free fluid was also seen in the abdomen, the density of which was high [Figures 1–3]. With this constellation of findings, a diagnosis of ruptured double fetus-in-fetu (FIF) was made and exploratory laparotomy was performed.

**Figure 4:**
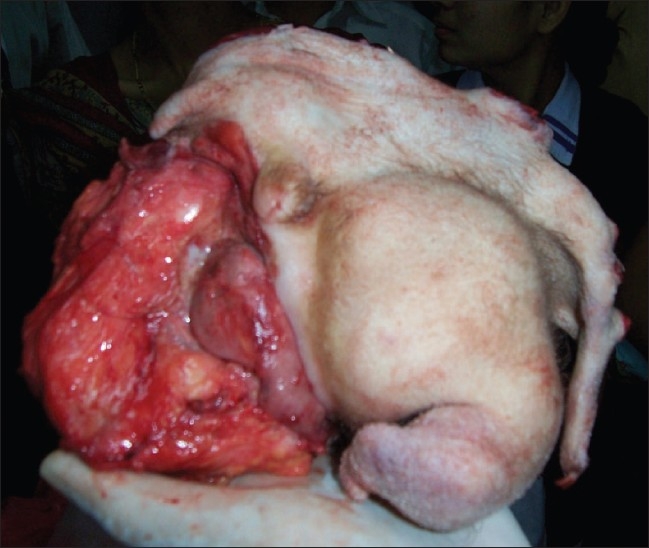
The surgical specimen

**Figure 5:**
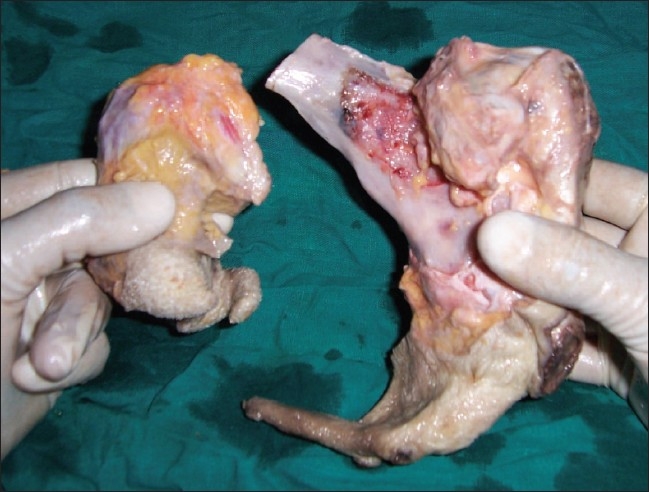
Excised surgical specimen demonstrates two incompletely developed fetuses

On laparotomy, a 15-cm encapsulated retroperitoneal mass with a broad vascular pedicle (attached to the retroperitonium below the aortic bifurcation) was found. The mass was resected *en bloc*; however, the capsule was found ruptured at places. On removing the capsular covering of the resected surgical specimen, it was found covered with hairy skin and a cheesy, pultaceous material; there were rudimentary limbs showing nails [Figure 4]. The excised specimen was cut open, and removal of the overlying fat and soft tissue material revealed two anencephalic malformed fetuses within the mass, with one fetus showing many matured bony elements, including the spine (the most defining element) and extremities; the other fetus was comparatively less developed, with a smaller vertebral column and small flipper-like rudimentary lower limbs [Figure 5]. These findings were consistent with two incompletely developed fetuses in the teratoma, confirming the diagnosis of twin FIF. The diagnosis was also confirmed on histopathology. The patient has been on follow-up for a year and is symptom free, with no evidence of recurrence.

## Discussion

FIF is a rare congenital entity in which a nonviable parasitic fetus grows within the body of its twin. This term was first coined by Meckel in the 18^th^ century and the first case was reported by Young in 1809. The incidence is about 1 in 500,000 births and only about 100 cases of FIF have been reported to date.[2]

Many theories have been postulated to explain the exact pathogenesis of FIF. The most commonly accepted theory is that it results from an abnormal diamniotic-monozygotic twin pregnancy in which a smaller cell mass is included within a maturing sister embryo due to unequal division of the totipotent inner cell mass of the developing blastocyst. According to the next most popular theory, it may be a highly differentiated form of dermoid cyst, which itself is a highly differentiated form of a mature teratoma.[3]

FIF usually presents as a fetiform osseous / calcified mass, often in the abdomen of its host, with the retroperitoneum being the most common site to be affected; however, the cranial cavity, pelvis, scrotal sac, sacrococcygeal region, mesentery, right iliac fossa, and oral cavity are also affected rarely. The condition usually presents in childhood. FIF is usually single, though as many as five FIFs have been reported by Kimmel *et al*., who described a case of a newborn with a cerebral tumor containing five human fetuses. Although, an FIF may increase in size and cause local mass effect and hemorrhage, rupture is not common.[3,4]

FIF is usually seen as a well-defined complex mass enclosed within a capsule, which is usually an amnion-like membrane. Identifiable bones and fat may be seen on plain films, but these findings are more vividly demonstrated with CT scan or MR studies. In addition, the vascular pedicle may be detected on CT scan.[5] On USG, it is seen as a mass with a complex echo pattern, with fluid, soft tissue, and calcification all being often identified within it.[6]

FIF usually resembles a poorly formed acardiac / anencephalic twin. The contents of the mass may vary; for example, it may contain extremity bones, pelvic bones, ribs, thoracic and abdominal organs, eyes, ears, mouth, skin, hair, nails, etc. Willis has emphasized that the identification of the vertebral column secures the diagnosis of FIF and differentiates this entity from teratoma.[7] Occasional cases have been reported in which a spinal column could not be identified on imaging. In addition to the presence of spinal elements with limb buds, other features distinguishing this condition from teratoma are its embryological origin, its invariable benignity, and the underdeveloped (though at times well-developed) organ systems within it. In our case, two distinct vertebral columns were detected in the mass on CT scan itself, which confirmed the diagnosis of twin (double) FIF.[3,7,8]

On, antenatal USG, FIF usually presents as a complex mass in the fetal abdomen. The general appearance is of a well-delineated, encapsulated, echogenic mass suspended in or partially surrounded by fluid. Occasionally, the diagnosis may be easily made if the rudimentary spine is recognized.[8]

In conclusion, FIF is a rare clinical entity and rarer still is a double FIF. The diagnosis can be accurately made by CT imaging. The demonstration of spinal elements is the most defining element of this condition.
